# Risk factors and effect on mortality of superinfections in a newly established COVID-19 respiratory sub-intensive care unit at University Hospital in Rome

**DOI:** 10.1186/s12890-023-02315-9

**Published:** 2023-01-20

**Authors:** Alessandra Iacovelli, Alessandra Oliva, Guido Siccardi, Angela Tramontano, Daniela Pellegrino, Claudio Maria Mastroianni, Mario Venditti, Paolo Palange

**Affiliations:** 1 Department of Public Health and Infectious Diseases, Sapienza University of Rome, Rome, Italy; 2 Pulmonology Respiratory and Critical Care Unit, Policlinico Umberto I Hospital Rome, Rome, Italy; 3 Department of Public Health and Infectious Diseases, Sapienza University of Rome, Rome, Italy; 4 Infective Diseases Unit, Policlinico Umberto I Hospital Rome, 00185 Rome, Lazio Italy

**Keywords:** COVID-19, Superinfections, MDR pathogens, Sub-intensive care unit, *Acinetobacter baumannii*, CAPA

## Abstract

**Background:**

Little is known on the burden of co-infections and superinfections in a specific setting such as the respiratory COVID-19 sub-intensive care unit. This study aims to (i) assess the prevalence of concurrent and superinfections in a respiratory sub-intensive care unit, (ii) evaluate the risk factors for superinfections development and (iii) assess the impact of superinfections on in-hospital mortality.

**Methods:**

Single-center retrospective analysis of prospectively collected data including COVID-19 patients hospitalized in a newly established respiratory sub-intensive care unit managed by pneumologists which has been set up from September 2020 at a large (1200 beds) University Hospital in Rome. Inclusion criteria were: (i) COVID-19 respiratory failure and/or ARDS; (ii) hospitalization in respiratory sub-intensive care unit and (iii) age > 18 years. Survival was analyzed by Kaplan–Meier curves and the statistical significance of the differences between the two groups was assessed using the log-rank test. Multivariable logistic regression and Cox regression model were performed to tease out the independent predictors for superinfections’ development and for mortality, respectively.

**Results:**

A total of 201 patients were included. The majority (106, 52%) presented severe COVID-19. Co-infections were 4 (1.9%), whereas 46 patients (22%) developed superinfections, mostly primary bloodstream infections and pneumonia. In 40.6% of cases, multi-drug resistant pathogens were detected, with carbapenem-resistant *Acinetobacter baumannii* (CR-Ab) isolated in 47%. Overall mortality rate was 30%. Prior (30-d) infection and exposure to antibiotic therapy were independent risk factors for superinfection development whereas the development of superinfections was an independent risk factors for in-hospital mortality. *CR-Ab* resulted independently associated with 14-d mortality.

**Conclusion:**

In a COVID-19 respiratory sub-intensive care unit, superinfections were common and represented an independent predictor of mortality. CR-Ab infections occurred in almost half of patients and were associated with high mortality. Infection control rules and antimicrobial stewardship are crucial in this specific setting to limit the spread of multi-drug resistant organisms.

**Supplementary Information:**

The online version contains supplementary material available at 10.1186/s12890-023-02315-9.

## Introduction

As of 28th July 2022, more than 570,000,000 people had been infected with SARS-CoV-2 worldwide, with more than 6,000,000 deaths [1]. Around 10% need hospitalization; respiratory failure, hyperinflammatory response, thrombotic events and superinfections are possible complications [2].

Viral respiratory illnesses predispose patients to bacterial and fungal infections; while this link has been widely described in relation with influenza pneumonia, it is still unclear the exact roles co-pathogens play in patients with COVID-19 [3]. Indeed, data available from the flu pandemics showed how bacterial co-infections and superinfections have a worse outcome than either infection on its own [4], leading the scientific community to consider antibiotic administration in patients admitted for influenza pneumonia [5].

During the first wave of COVID-19 pandemic, although few papers reported a low incidence of bacterial co-infections [6], most patients received antibiotics at disease onset [7, 8]. Later on, questions began to be asked on the relation between SARS-CoV-2 and co-pathogens [9, 10]. A living review ensuring regular updates every three months shows that co-infections and superinfections are encountered respectively in 4.9% and 16% of patients hospitalized for COVID-19 [11].

While the rate of co-infections has an important role in deciding whether to use empiric antibiotic therapy, superinfection rate tends to be higher in patients with prolonged hospital stay, worst clinical presentation, and need for Intensive Care Unit (ICU) [11]. Wide spectrum antibiotic use and reduced adherence to infection prevention practices contributed to a rapid spread of multidrug-resistant bacteria (MDR) [12, 13], which increase during hospital stay and represent over 70% of the isolates after 30 days of hospitalization [14].

In most centers in Italy, COVID-19 patients were admitted to ICU or ordinary wards based on their clinical conditions. Italian hospitals faced an unpreceded massive inflow in a short period of time [15]. In centers with more of 500 beds, semi-intensive units were set up, with integrated management between emergency medicine specialists, infectious diseases specialists, pulmonologists, and other specialties. In our Academic Hospital in Rome, a center with more than 1200 beds, starting from September 2020, a semi-intensive unit was set up managed by pneumologists.

To our knowledge, little is known about the prevalence of concurrent and superinfections in this peculiar setting.

Based on these premises, we carried out a study specifically targeting patients hospitalized in a respiratory sub-intensive care unit with the aims to (i) assess the prevalence and etiology of co- and superinfections, (ii) evaluate the risk factors for superinfection development and (iii) assess the impact of superinfections on in-hospital mortality.

## Materials and methods

### Study design

We performed a single-center, retrospective analysis of prospectively collected data on patients with COVID-19 pneumonia hospitalized in a respiratory sub-intensive respiratory care unit at Azienda Ospedaliero-Universitaria Policlinico Umberto I, Sapienza University of Rome, from November 2020 for the following 6 months, until April 2021.

Inclusion criteria were: (i) diagnosis of COVID-19 respiratory failure and/or acute respiratory distress syndrome (ARDS), (ii) hospitalization in a sub-intensive respiratory care unit for > 48 h and (iii) age > 18 years. Exclusion criteria included: age < 18 years, hospitalization in a sub-intensive respiratory care unit for < 48 h and missing data.

Diagnosis of SARS-CoV-2 infection was made with molecular analyses. Diagnosis of respiratory failure was made on clinical presentation and arterial blood gases (ABGs), a lung CT scan was performed to detect ground glass opacity and/or others lesions compatible with COVID-19 pneumonia. Acute respiratory distress syndrome was diagnosed on clinical and ABGs data according to Berlin definitions [16]. The decision to hospitalize in sub-intensive respiratory care unit was made on a clinical judgment by the emergency department or internal medicine doctors or infectious disease specialists in case of patients admitted from other wards.

### Setting

Starting from September 2020, a sub-intensive respiratory care unit with 42 beds managed by pneumologists was set up in our 1200-bed Academic Hospital. Patients were admitted in case of severe respiratory failure and/or ARDS due to COVID-19 pneumonia requiring oxygen therapy and/or Helmet continuous positive airway pressure (CPAP) treatment or non-invasive mechanical ventilation (NIMV) (severe disease and critical disease according to WHO definitions [17]). Oxygen therapy was administered with Venturi Mask or high flow nasal cannula (HFNC) or with Helmet CPAP treatment or NIMV. In case of patients with tracheostomy, invasive ventilation was performed. Ventilation was performed in pressure support modality or assisted-controlled modality.

Patients required the use of continuous vital signs monitoring, and, in most cases, central venous catheter (CVC) or arterial catheters’ placement, total parenteral nutrition and, in some cases, sedation. Sedation was performed in case of non-adaptation to ventilation. Drugs administered in case of sedation were midazolam or dexmedetomidine. The level of sedation was related to the possibility of maintaining non-invasive ventilation.

We managed also critically patients with septic shock, or heart failure or requiring renal replacement, in this case with integrated management with nephrology specialists. We collaborated with an infectious disease specialist to treat superinfections.

Criteria for ICU-transfer was: if clinical conditions worsened despite ventilation and clinical care, an anesthesiologist consultation was required, and patients were transferred to ICU in case orotracheal intubation was needed.

Main differences with ICU setting were: (i) we did not manage intubated patients; (ii) most patients were not sedated and (iii) we have a different number of nurses, with one nurse assisting almost height patients during the work shift (nurse/patient ratio 1:8); (iv) ICU was managed by anesthesiologists.

### Procedures

Nasopharyngeal swab samples were collected, and SARS-CoV-2 RNA was detected by using real time RT-PCR assay (RealStar SARS-CoV2 RT-PCR, Altona Diagnostics).

All patients received oxygen therapy and/or HFNC or CPAP or NIMV treatment based on respiratory failure severity.

### Clinical criteria for diagnostic cultures and infection management

Screening for multi-drug resistant (MDR) bacteria [methicillin-resistant *Staphylococcus aureus* (MRSA), vancomycin-resistant *Enterococcus faecium* (VRE), or carbapenem resistant (CR) gram-negative bacilli] colonization was systematically performed by means of rectal swab, at admission to the sub-intensive respiratory unit and every 7 days afterwards.

Respiratory samples included sputum and/or tracheobronchial aspirate (when feasible) and were collected on clinical bases, i.e., in the presence of augmented or modified respiratory secretions or when a pulmonary superinfection was suspected because of worsening of respiratory changes, with or without fever.

Additional microbiological procedures (i.e., blood or urine cultures) were performed during hospitalization in case of suspected superinfections, as requested by the attending physicians or the Infectious Disease (ID) consultant. Tracheobronchial aspirate (TBA) galactomannan (GM) and fungal culture were performed based on clinical suspicion of *Aspergillus* spp superinfection.

In case of suspected superinfections, all patients received empiric antibiotic therapy whereas definitive antibiotic therapy was based on the results of cultures. The clinical approach to bacterial and fungal superinfections was managed by a dedicated ID physician (author name, AO).

### Microbiological methods

Isolated colonies from biological samples were identified by the Matrix-Assisted Laser Desorption Ionization–Time Of Flight Mass Spectrometry (MALDI-TOF MS) system (Bruker Daltonik GmbH, Bremen, Germany). Antimicrobial susceptibility tests were performed with Vitek 2 automated system (bioMérieux, Marcy l’Etoile, France) or with the MicroScan system (Beckman Coulter, Brea, CA, USA), according to the manufacturer’s instructions.

Fungal cultures were incubated for 7 days at 30 °C on Sabouraud selective media, whereas GM test in serum and TBA was performed according to manufacturer’s instructions (Platelia Aspergillus EIA, Bio-Rad).

### Definitions

Respiratory failure was diagnosed in case of a value of PaO_2_ < 60 Torr at room air at ABGs, whereas PaO_2_/FiO_2_ ratio (P/F ratio) was used as an indicator of severity, according to Berlin definitions [16]. PaO_2_/FiO_2_ was recollected at hospital admission and daily. Only P/F at hospital admission was included into statistical analysis. COVID-19 pneumonia was diagnosed by clinical data, ABGs and lung CT scan performed for all patients at hospital admission [8]. Severe and critical disease were defined according to WHO definitions [17].

Prior (30-d) infections referred to infections diagnosed within 30 days before hospital admission; prior (30-d) antibiotic exposure included the receival of antibiotic therapy in the 30 days before diagnosis of secondary infections.

Co-infections were defined as infection onset less than 48 h from admission or present at hospital admission whereas superinfections were diagnosed by clinical signs of infections and a positive culture on blood, urine, and respiratory specimens, after 48 h from hospital admission [11, 18]. MDR and extensively drug-resistant pathogens (XDR) were defined based on susceptibility to different classes of antibiotics [19].

Pulmonary Aspergillosis following COVID-19 was defined according to the recently proposed definition [20].

Infections were defined according to the standard definitions of the ECDC [21]. The likely or ascertained source of infection was indicated by the attending physician or by the ID consultant in the medical record and defined in accordance with guidelines [21, 22]. Primary bloodstream infection (BSI) was defined as BSI occurring in patients without a recognized source of infection. Catheter-related related BSI (CR-BSI) was defined if the semiquantitative culture of the catheter tip was positive for the same microorganism isolated from the blood [23].

Mortality referred to in-hospital death for all causes, while 14-d survival estimates were performed in patients with superinfections.

The study was conducted according to the guidelines of the Declaration of Helsinki. The study was approved by the local Ethics Committee of Azienda Ospedaliera Universitaria Policlinico Umberto I, Rome (ID Prot. 109/2020). Given the retrospective nature of the study, specific informed consent was waived. However, at hospital admission, all the patients provided general consent for the use of their clinical data in future studies.

### Statistical analysis

The data were given as medians with interquartile ranges (IQR) for continuous variables and as simple frequencies, proportions, and percentages for categorical variables. Mann–Whitney test was used for unpaired samples. Dichotomous variables were compared using Fisher's exact tests or chi-square test statistics, as appropriate. Survival was analyzed by Kaplan–Meier curves and the statistical significance of the differences between the 2 groups was assessed using the log-rank test. Multivariable logistic regression and Cox regression model were performed to tease out the independent predictors for superinfections’ development and for mortality, respectively. All statistical analyses were performed with STATA/IC (StataCorp, version 15).

## Results

### General population

A total of 201 patients were included in the study, with a median age of 67 years (IQR 56–80); 106 (52%) presented with severe COVID-19. General features of the study population are shown in Table 1.

**Table 1:** Baseline characteristics of patients and type of superinfections

Variable	Total population N = 201	Non infection group N = 155	Infection group N = 46	*p* value
Age, median (IQR), years	67 (56–80)	66 (55–77)	77 (65–83)	**0.0005**
Sex (M), n (%)	127 (63)	100 (64)	27 (58%)	0.49
*Demographics*				
Autoimmune disease, n (%)	3 (1.4)	2 (1.2)	1 (2.1)	0.54
Chronic steroid/immunosuppressive therapy, n (%)	17 (8.4)	9 (5.8)	8 (17.3)	**0.029**
Diabetes, n (%)	45 (22)	29 (18.7)	16 (34.7)	**0.027**
Chronic ischemic heart disease, n (%)	43 (21)	24 (15.4)	19 (41.3)	**0.0004**
Hypertension, n (%)	93 (46)	67 (43.2)	26 (56.5)	0.13
Heart failure, n (%)	21 (10.4)	8 (5.1)	13 (28.2)	**0.0001**
Atrial fibrillation, n (%)	24 (11.9)	14 (9)	10 (21.7)	**0.034**
Vasculopathy, n (%)	19 (9.4)	15 (9.6)	4 (8.6)	0.99
Cerebrovascular disease, n (%)	12 (5.9)	7 (4.5)	5 (10.8)	0.15
Dementia, n (%)	15 (7.4)	9 (5.8)	6 (13)	0.10
COPD, n (%)	30 (14.9)	22 (14.1)	8 (17.3)	0.63
Chronic hepatopathy, n (%)	9 (4.4)	5 (3.2)	4 (8.6)	0.21
Solid malignancy, n (%)	17 (8.4)	9 (5.8)	8 (17.3)	**0.029**
Hematological malignancy, n (%)	7 (3.4)	5 (3.2)	2 (4.3)	0.66
Chronic kidney disease, n (%)	13 (6.4)	7 (4.5)	4 (8.6)	0.27
Obesity (BMI > 30), n (%)	31 (15.4)	26 (16.7)	5 (10.8)	0.48
Charlson Comorbidity Index, median (IQR)	3 (2–5)	3 (1–4)	5 (3.25–6)	**0.0001**
Prior (30-d) infections*, n (%)	13 (6.4)	4 (2.5)	9 (19.5)	**0.0003**
Prior (30-d) chemotherapy, n (%)	2 (0.9)	1 (0.6)	1(2.1)	0.4
Prior (30-d) hospitalization, n(%)	27 (13.4)	17 (10.9)	10 (21.7)	0.24
Central line catheter, n (%)	12 (5.9)	3 (1.9)	9 (19.5)	**0.0001**
Total parental nutrition, n (%)	12 (5.9)	3 (1.9)	9 (19.5)	**0.0001**
Severe COVID-19**, n (%)	106 (52.7)	76 (49)	30 (66)	0.06
First PaO_2_/FiO_2_ ratio at ED admission, median (IQR)	300 (248–333)	305 (252–338)	271 (213–320)	0.06
Transferred from ICU, n (%)	2 (0.9)	1 (0.6)	1(2.1)	0.4
*Symptoms at COVID onset, n (%)*				
Fever	143 (59)	112 (72)	31 (67.3)	0.57
Cough	66 (32)	59 (38)	7 (15.2)	0.039
Dyspnea	114 (56)	86 (55.4)	28 (60.8)	0.6
*Laboratory parameters at admission*				
Leucocytes (× 10^3^/mm^3^), median (IQR)	7050 (4905–10,590)	7110 (5120–10,150)	6900 (4570–11,410)	0.9
Neutrophils (× 10^3^/mm^3^), median (IQR)	5585 (3660–8990)	5580 (3660–8640)	5610 (3690–9640)	0.79
Lymphocyte (× 10^3^/mm^3^), median (IQR)	760 (532,5–1020)	760 (540–1040)	690 (490–990)	0.49
Platelet (× 10^3^/mm^3^), median (IQR)	195 (156.25–256.75)	196 (1257–256)	190 (151–270)	0.98
Creatinine, median (IQR) mg/dl	1 (0.8–1.2)	0.9 (0.8–1.1)	1.1 (0.9–1.4)	0.075
Albuminemia, median (IQR) g/dl	3.7 (3.5–4)	3.7(3.5–4)	3.7 (3.1–3,9)	0.9
D-Dimer, median (IQR) U/l	760 (473.2–1369.75)	665.5 (431–1102)	1281 (756–2335)	0.0001
CRP, median (IQR) mg/dl	5.4 (1.7–10)	5.5 (2–10.3)	5.7 (1.2–10.4)	0.65
MDR colonization, n (%)	23 (11)	5 (3.2)	18 (39)	**0.0001**
Long of in-hospital stay, median (IQR), days	18 (12–26)	16 (11–22)	30 (19–45)	**0.001**
Prior antibiotic therapy (30-d), n (%)	162 (80.5)	116 (74)	46 (100)	**0.0001**
Transfer to ICU	12 (5.9)	5 (3.2)	7 (15.2)	0.068
Corticosteroid therapy, n (%)	195 (97)	149 (96.1)	46 (100)	0.33
Mortality, n (%)	61 (30.3)	35 (22)	26 (56)	**0.0001**
Pronation, n (%)	17 (8.5)	15 (9.7)	2 (4.3)	0.254
Sedation, n (%)	16 (8)	10 (6.4)	6 (13)	0.155
Respiratory failure treatment, n (%)				**0.008**
HFNC	25 (12.4)	19 (12.2)	6 (13)	
Venturi mask	51 (25.4)	41 (26.4)	10 (21.7)	
Helmet CPAP	104 (51.7)	85 (54.8)	19 (41.3)	
NIMV	21 (10.4)	10 (6.4)	11 (23.9)	
Superinfections episodes°, n (%)	64 (100)	NA	64 (100)	NA
*Type of superinfections episodes, n (%)*				
Primary bloodstream infection	23 (35.9)	NA	23 (35.9)	NA
Hospital-acquired pneumonia	19 (29.6)	NA	19 (29.6)	NA
Urinary infections	18 (28.5)	NA	18 (28.5)	NA
CR-BSI	2 (3.1)	NA	2 (3.1)	NA
Skin and soft tissues infections	1 (1.5)	NA	1 (1.5)	NA
*Clostridoides difficile* colitis	1 (1.5)	NA	1 (1.5)	NA

Bold is used to emphasize relevant *p* values

COPD: chronic obstructive pulmonary disease; ED: Emergency department; CRP: C-reactive protein; MDR: multi-drug resistant; ICU: intensive care unit; CR-BSI: catheter-related bloodstream infection. NA: not applicable. 30-d: 30 days before infection development

*Prior (30-d) infections referred to infections diagnosed within 30 days before hospital admission

**Severe COVID-19 was defined according to WHO definitions [17]

°Superinfections episodes: the total number is different because several patients experienced > 1 infective episode (46 patients for a total of 64 episodes). HFNC: High Flow Nasal Cannula; CPAP: Continuous Positive Airway Pressure; NIMV: Non-invasive mechanical ventilation

The majority of patients (156, 77%) was admitted to our sub-intensive care unit directly from the emergency room, 43 (21%) from ordinary wards because of respiratory worsening and only 2 (0.9%) from ICU. Twelve patients (5.9%) were transferred to ICU. Co-infections were 4 (1.9%), all due to *Mycoplasma pneumoniae* whereas 46 patients (22%) developed a superinfection. Overall mortality rate was 30%.

Study population was further divided into two groups according to the development of superinfection [Infection (n = 46, 22%) and No-infection (n = 155, 78%) groups, respectively].

#### Comparison between survivors and non-survivors

As shown in Table 2, patients who died presented higher prevalence of comorbidities, and, consequently, a higher CCI than survivors (p = 0.0001). Moreover, non-survivors presented a higher percentage of infection in last 30-d before sub-intensive care unit admission (14.9% vs 2.8%, p = 0.036), severe COVID-19 at presentation (72% vs 44%, p = 0.004) and colonization by MDR pathogens (24% vs 5.7%, p = 0.004). At hospital admission, non-survivors presented a lymphocytes count, platelets count and albuminemia significantly lower (p = 0.001, 0.004 and 0.0033, respectively) and C-reactive protein, D-dimer, and creatinine significantly higher than patients who survived (p = 0.02, 0.0022 and 0.02, respectively). The development of superinfections was more common in non-survivors (42% vs 14%, p = 0.0001). Likewise, sedation use was more frequent in non-survivors (22% vs 2.1%, p < 0.0001). Non-survivors were treated more frequently with NIMV and less commonly with venturi mask (29.5% vs 2.1% and 9.8% vs 32%, respectively).

**Table 2:** Characteristics of survivors and non-survivors

Variable	Non-survivors N = 61	Survivors N = 140	*p* value
Age, median (IQR), years	82 (73–87)	61 (53–72)	**0.0001**
Sex (M), n (%)	36 (59.4)	91 (65)	0.43
*Demographics*			
Chronic steroid/immunosuppressive therapy, n (%)	4 (6.5)	13 (9.2)	0.59
Diabetes, n (%)	19 (31.1)	26 (18.5)	**0.0008**
Chronic ischemic heart disease, n (%)	27 (44)	16 (11.4)	**0.0001**
Hypertension, n (%)	37 (60.6)	56 (40)	**0.008**
Heart failure, n (%)	16 (26.2)	5 (3.5)	**0.0001**
Atrial fibrillation, n (%)	17 (27.8)	7 (5)	**0.0001**
Vasculopathies, n (%)	15 (24.5)	4 (2.8)	**0.0001**
COPD, n (%)	12 (19.6)	18 (12.8)	0.28
Solid malignancy, n (%)	12 (19.6)	5 (3.5)	**0.0001**
Hematological malignancy, n (%)	1 (1.6)	6 (4.2)	0.99
Chronic kidney disease, n (%)	7 (11.4)	6 (4.2)	**0.01**
Obesity (BMI > 30), n (%)	7 (11.4)	24 (17.1)	0.39
Charlson CI, median (IQR)	5 (4–6)	2 (1–4)	**0.0001**
Prior (30-d) infections°, n (%)	9 (14.7)	4 (2.8)	**0.0036**
Prior hospitalization, n (%)	14 (22.8)	13 (9.2)	**0.0129**
Central line catheter, n (%)	9 (14.7)	3 (2.1)	**0.0009**
Total parental nutrition, n (%)	10 (16.3)	2 (1.4)	**0.0001**
Severe COVID, n (%)	44 (72.1)	62 (44.2)	**0.004**
PaO2/FiO2 ratio, median (IQR)	248 (210–310)	310 (271–343)	**0.0001**
Transfer from ICU, n (%)	0 (0)	2 (1.4)	0.345
Transfer to ICU, n (%)	10 (16.4)	2 (1.4)	< 0.0001
Prior (30-d) antibiotic therapy, n (%)	54 (88.5)	108 (77.1)	0.08
MDR colonization, n (%)	15 (24.5)	8 (5.7)	**0.0004**
Superinfection, n (%)	26 (42.6)	20 (14.2)	**0.0001**
Corticosteroid therapy for SARS-CoV2, n (%)	61 (100)	134 (95)	0.18
Pronation, n (%)	5 (8.2)	12 (8.6)	0.930
Sedation, n (%)	13 (22.0)	3 (2.1)	** < 0.0001**
*Respiratory failure treatment, n (%)*			
HFNC	7 (11.5)	18 (12.9)	**< 0.0001**
Venturi mask	6 (9.8)	45 (32.1)	
CPAP	30 (49.2)	74 (52.9)	
NIMV	18 (29.5)	3 (2.1)	
*Laboratory parameters at admission*			
Leucocytes (× 10^3^/mm^3^) median (IQR)	6865 (4485–10,732)	7050 (5072–9547)	0.85
Neutrophile (× 10^3^/mm^3^) median (IQR)	5535 (3682–9890)	5585 (3607–8275)	0.53
Lymphocyte (× 10^3^/mm^3^) median (IQR)	555 (427–875)	840 (642–1077)	**0.0001**
Platelet (× 10^3^/mm^3^) median (IQR)	171 (138–232)	198,5 (163–261)	**0.04**
Creatinine, median (IQR) mg/dl	1.2 (0.86–1.5)	0.9 (0.8–1.09)	**0.02**
Albumin, median (IQR) g/dl	3.6 (3.3–3.8)	3.8 (3.5–4)	**0.0033**
D-dimer, median (IQR) U/l	1179 (735–1894)	624 (433–1109)	**0.0022**
CRP, median (IQR) mg/dl	8,375(3.3–13)	4.2 (1.4–9)	**0.02**

Bold is used to emphasize relevant *p* values

COPD: chronic obstructive pulmonary disease; Charlson CI: Charlson Comorbidity Index; P/F ratio: PaO_2_/FiO_2_ ratio; CRP: C-reactive protein; MDR: multi-drug resistant; ICU: intensive care unit. 30-d: 30 days before infection development. HFNC: High Flow Nasal Cannula; CPAP: Continuous Positive Airway Pressure; NIMV: Non-invasive mechanical ventilation. °: Prior (30-d) infections referred to infections diagnosed within 30 days before hospital admission

At multivariable analysis, independent risk factors for in-hospital mortality were the development of superinfections [OR 3.04 (1.16–7.96), p = 0.024], age > 65 years [OR 5.25 (1.36–19.84), p = 0.014], CCI > 5 [OR 4.81 (1.81–12.71), p = 0.002], lymphocytes count < 750/mmc [OR 3.08 (1.21–7.84), p = 0.018], use of sedation [OR 6.68 (1.36–32.71), p = 0.019] and NIMV [OR 11.81 (2.04–68.16), p = 0.002] (Table 3).

**Table 3:** Multivariate analysis of factors associated with in-hospital mortality

	OR (95% CI)	*p* value
Superinfections	3.04 (1.16–7.96)	**0.024**
Age > 65 years	5.25 (1.36–19.84)	**0.014**
Severe COVID-19	2.09 (0.83–5.26)	0.116
CCI > 5	4.81 (1.81–12.71)	**0.002**
Lymphocytes < 750/mmc	3.08 (1.21–7.84)	**0.018**
Sedation	6.68 (1.36–32.71)	**0.019**
*Respiratory failure treatment*		
Venturi mask (ref)	1	**–**
HFNC	2.38 (0.49–11.60)	0.281
CPAP	1.92 (0.54–6.78)	0.308
NIMV	11.81 (2.04–68.16)	**0.006**

Bold is used to emphasize relevant *p* values

CCI: Charlson Comorbidity Index, HFNC: High Flow Nasal Cannula, CPAP: Continuous Positive Airway Pressure, NIMV: Non-invasive mechanical ventilation

#### Comparison between infection and no-infection groups

Patients with superinfections presented a higher rate of severe COVID-19 on admission, albeit not significant (66% vs 49%, p = 0.6), were older [77 (65–83) vs 66 (55–77) years, p = 0.0005] and had a higher rate of diabetes, chronic ischemic heart disease, chronic heart failure, atrial fibrillation and malignancy, with a higher Charlson Comorbidity Index (CCI) (p = 0.0001) (Table1). The number of patients coming from or transferred to the ICU did not differ between the two groups.

Chronic corticosteroids therapy (17% vs 5%, p = 0.029) and antibiotic exposure in the previous 30-d before superinfections development (100% vs 74%, p = 0.0001) were more common in the Infection group. Given the severity of COVID-19 pneumonia, corticosteroid treatment was similar in both groups (100% vs 96.1%, p = 0.33). Colonization with MDR pathogens was higher in the group of patients with super-infections (39% vs 3.2%, p = 0.0001). No differences were observed for pronation and sedation use in the two groups, whereas NIMV was more frequent in patients developing superinfections (23.9% vs 6.4%).

Mortality rate was higher in patients with superinfections (56% vs 22%, p = 0.0001). Furthermore, they presented a longer in-hospital stay [30 (19–45) vs 16 (11–22) days, p = 0.001].

At multivariable analysis, prior (30-d) infection [OR 3.95 (1.00–15.7), p = 0.05] and exposure to antibiotic therapy in last 30-d [OR 4.82 (1.28–18.1), p = 0.020] were independent risk factors for new onset superinfections development (Table 4).

**Table 4:** Multivariate analysis evaluating independent risk factors associated with the onset of superinfections

	OR (95% CI)	*p* value
Age > 65 years	1.6 (0.60–4.56)	0.322
Charlson CI > 5	2.28 (0.89–5.83)	0.084
Prior infections (30-d)*	3.95 (1.00–15.7)	**0.050**
Sedation	1.58 (0.43–5.7)	0.487
Severe COVID-19	1.46 (0.66–3.25)	0.346
Prior antibiotic therapy (30-d)**	4.82 (1.28–18.1)	**0.020**
*Respiratory failure treatment*		
Venturi mask (ref)	1	–
HFNC	1.27 (0.34–4.66)	0.717
Helmet CPAP	0.69 (0.25–1.92)	0.488
NIMV	2.12 (0.55–8.12)	0.271

Bold is used to emphasize relevant *p* values

Charlson CI: Charlson Comorbidity Index; HFNC: High Flow Nasal Cannula; CPAP: Continuous Positive Airway Pressure; NIMV: Non-invasive mechanical ventilation

*Prior (30-d) infections referred to infections diagnosed within 30 days before hospital admission

**Prior (30-d) antibiotic exposure included the receival of antibiotic therapy in the 30 days before diagnosis of secondary infections

#### Subjects with superinfections

Overall, 46 patients (22%) developed superinfections, with the different causative pathogens shown in Supplementary Table 1. Several patients experienced > 1 infective episode and therefore we recorded 64 infections. Primary BSI was the most frequent superinfection with 23 episodes (35.9%), followed by pneumonia (29.6%), urinary infections (28.5%), CR-BSI (3.1%), skin/soft tissue infections and *Clostridioides difficile* infection (1.5% each).

First infective episode was diagnosed after a median of 14.5 days (IQR 7–22) from sub-intensive unit admission. In 23 cases (40.6%), MDR pathogens were detected, with CR *Acinetobacter baumannii* (CR-Ab) isolated in 11 (47%) of them. Others MDR involved were MRSA, VRE and CR *Klebsiella pneumoniae* (CR-Kp) (6 cases, 21.5%; 4 cases, 17.3% and 2 cases, 13% respectively). All the strains of *A. baumannii* were resistant to meropenem; all but one exhibited colistin susceptibility. Cefiderocol susceptibility was available only for 6 strains; all the tested strains were susceptible at disk diffusion method (Supplementary Table2).

**Fig. 1:**
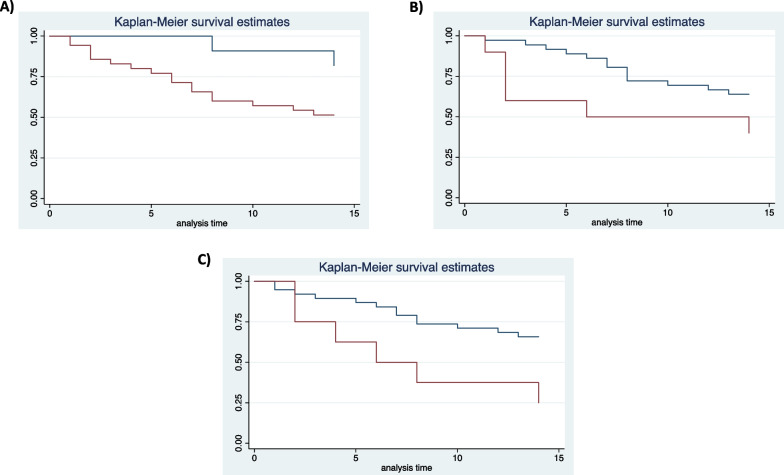
14-d survival estimates in patients with superinfections according to age (**a** age > 65-y, red line; age < 65-y blue line), severity of infection (**b** presence of septic shock, red line; absence of septic shock, blue line) and causative agent (**c** XDR-Ab, red line; pathogens other than XDR-Ab, blue line)

Kaplan–Meier survival curves at 14 days from the superinfection showed different rates of mortality in patients with age > 65 years, septic shock at infection onset and XDR *A. baumannii* as etiologic agents of first infective episode (Fig. 1a–c).

At multivariable analysis, only XDR *Acinetobacter baumannii* resulted independently associated with 14-d mortality [OR 5 (1.3–19.1), p = 0.018] (Table 5).

**Table 5:** Multivariate analysis of risk factors associated with mortality at 14-d from infection onset

	HR (95% CI)	*p* value
CCI > 5	1.1 (0.3–3.6)	0.87
Severe COVID	1.5 (0.5–4.6)	0.44
Female sex	0.2 (0.05–0.7)	**0.021**
Septic shock	2 (0.5–6.9)	0.255
First infective episode due to XDR *A. baumannii*	5 (1.3–19.1)	**0.018**
Age > 65 years	3.6 (0.5–22)	0.162

Bold is used to emphasize relevant *p* values

CCI: Charlson Comorbidity Index; XDR: extended drug resistant

## Discussion

This study presents the prevalence and the clinical impact of co-infections and superinfections in COVID-19 patients hospitalized in a respiratory sub-intensive care unit. We showed that (i) co-infections were uncommon while the development of superinfections occurred in almost one quarter of patients and were independently associated with mortality; (ii) MDR organisms were isolated in approximately 40% of patients, with CR-Ab accounting for almost half of cases and (iii) CR-Ab as the causative agent of the first infective episode was associated with early (14-d) mortality.

To the best of our knowledge, the prevalence and characteristics of superinfections in COVID-19 patients hospitalized in a respiratory sub-intensive care unit are not well understood and represent a significant knowledge gap in literature data.

Indeed, from the spread of SARS-CoV-2 pandemic, several studies reported the rates of bacterial, viral and/or fungal infections in COVID-19 patients, especially in ICU. A study conducted during the first pandemic wave reported a high rate of incidence and prevalence of co-infections during COVID-19 [24]. This argument seemed to be consistent with previously demonstrated association between other viral infections, such as influenza, and bacterial co-infections, especially with *Staphylococcus aureus* [25], thus justifying the use of antimicrobials. Under these circumstances, in the clinical practice also COVID-19 patients had been routinely treated with empirical antibiotic therapy, especially in case of critical illness during 2020.

Nevertheless, according to the following literature [11–26] co-infections during COVID-19 were considered uncommon. Our report confirmed a low rate of bacterial co-infection (1.9%), with *Mycoplasma pneumoniae* as the etiologic agent in all the cases, although concerns were raised about diagnosing *M. pneumoniae* infection in COVID-19 patients only by means of serology diagnostic testing. These data are in accordance with our previous data collection referred to the first pandemic wave in which *Mycoplasma pneumoniae* was responsible for the 1.1% of co-infections [27] and sustain, in line with the literature and according to antimicrobial stewardship principles, [28] the possibility of an antibiotic sparing approach in the early phase of COVID-19 pneumonia.

Overall, 46 patients (22%) developed superinfections. Our data seems in line with literature [29, 30] in terms of development of superinfections. Indeed, recent reports showed an overall prevalence of bacterial superinfections of 16% in COVID-19 patients [31], ranging from 4.8 to 42.8%, with the highest prevalence in critically ill patients.

Chong et al. reported an overall time to diagnose pulmonary superinfections of 10 days (2–21 days) from initial hospitalization and 9 days (4–18 days) after ICU admission [32]. Likewise, in our study, the first infective episode was diagnosed after a median of 14.5 days from sub-intensive unit admission.

COVID-19 severity could in part explain the association between critical conditions and superinfections. Ripa M et al. concluded that factors associated with superinfections were low baseline lymphocyte count, baseline P/F ratio, and ICU admission [33], while Falcone and colleagues showed that invasive mechanical ventilation, carbapenem-resistant Enterobacterales (CRE) intestinal colonization, immunomodulatory agents (tocilizumab/baricitinib), high C-reactive protein on admission and previous treatment with piperacillin/tazobactam were risks factors for bacterial and fungal superinfections [14]. We confirmed the association between superinfections development and a previous exposure to antibiotic therapy and prior infection. Conversely, although NIMV was more frequent in patients developing superinfections, at multivariable analysis the mode of respiratory failure treatment did not predict superinfections in our population.

We reported an overall mortality rate of 30%, with a higher rate in patients with superinfections, especially when sustained by CR-Ab. The development of superinfections represented an independent risk factor for in-hospital mortality, together with age > 65 years, CCI > 5, severe COVID-19 and a count of lymphocytes < 750/mmc on admission. In a previous study conducted in 24 ICUs, trends of clinical, ventilatory and laboratory parameters throughout the entire ICU stay showed different slopes between survivors and non-survivors [34]. Furthermore, in our study sedation and NIMV were associated with mortality. This data could be explained considering that sedation and NIMV were used when other treatments (i.e., Helmet CPAP) failed or respiratory failure worsened despite Helmet CPAP treatment, suggesting a more severe respiratory condition. As a matter of fact, in most cases patients treated with NIMV and sedation presented severe ARDS.

As reported for patients hospitalized in ICU [31–35], in the present report MDR pathogens including MRSA, VRE, CR-*A. baumannii* and CR-*K. pneumoniae* were detected in 40.6% of cases, with CR-Ab, a common colonizer in the ICU setting, isolated in almost half of cases. This prevalence could be explained by considering that patients hospitalized in our sub-intensive unit presented several risk factors usually associated with *A. baumannii* infections, such as prolonged hospitalization, need of intensive care, presence of devices, older age, prior colonization, and prior use of antibiotics [36].

MDR in Gram-negative bacilli represent a major threat to human health and significantly contribute to global deaths attributable to and associated with bacterial antimicrobial resistance [37]. With this regard, superinfections caused by MDR Enterobacterales, *Pseudomonas aeruginosa* and *Acinetobacter baumannii* have been increasingly reported during the hospital stays of patients with severe COVID-19 [38].

Indeed, a recent study by Falcone and colleagues during the first pandemic wave in Italy (March–April 2020) showed that MDR organisms caused 65.1% of superinfections, slightly higher than our report. Interestingly, the rate of MDR infections increased during the hospital stay, from 50% in the first 7 days up to 70.4% if hospitalized for > 30 days. Although the majority of MDR organisms were represented by CRE, the increase during hospitalization mostly regarded non fermenters such as *P. aeruginosa*, *A. baumannii* and *S. maltophilia* [14].

These findings are in line with other reports showing that more than 60% of patients with COVID-19 who had a bacterial infection carried a highly resistant organism [18]. Furthermore, emerging data from the United States Centers for Disease Control and Prevention suggests that pandemic has resulted in rising rates of antimicrobial resistance, including CR-Ab [39].

More recently, national Italian data evaluating superinfections caused by CRE in hospitalized patients with COVID-19 from March to December 2020 found that the majority of infections occurred in the ICU, although almost one third of episodes occurred in medical wards; however, no data on sub-intensive respiratory units was present. Thirty-day mortality was 33.3% [40].

In a previous study, we evaluated the impact of COVID-19 on MDR Gram-negatives BSIs in a single ICU: while before pandemic patients were more likely to present CR-Kp BSIs, after COVID-19 CR-Ab BSIs were more commonly observed [41]. Furthermore, CR-Ab colonization was associated with MDR *A. baumannii* infection in COVID-19 patients [13].

Many hospitals have reported MDR outbreaks in ICU during the COVID-19 pandemic, mostly caused by Gram-negative bacteria or *Candida auris* [42]. Carbapenem-resistant *Acinetobacter baumannii* seems to be the main pathogen involved, with several outbreaks reported worldwide [42, 43].

Another Italian study compared colonization and infection rates before and after COVID-19; authors found that in the COVID-19 period, the incidence rate ratios of colonization and infection with CR-Ab increased 7.5- and 5.5-fold, respectively. Genome sequencing showed that all CR-Ab strains belonged to the CC92/IC2 clonal lineage [44]

More recently, a systematic review and meta-analysis analyzed the impact of the COVID-19 pandemic on MDR organisms across different healthcare settings by providing data on antimicrobial resistance before or during the COVID-19 pandemic. Authors found that the pandemic was not associated with a change in the incidence or proportion of MRSA or VRE; conversely, although not significant, an increase of resistant Gram-negatives (i.e., ESBL, CRE, MDR or carbapenem-resistant *Pseudomonas aeruginosa* or *Acinetobacter baumannii*) was observed, particularly in settings where enhanced infection control policies and/or antimicrobial stewardship programs were not reported. Furthermore, there was a small increase in the proportion of infections due to resistant *Acinetobacter spp* [18].

The apparent increase in incidence of Gram-negative, but not Gram-positive, antimicrobial resistance suggests that antibiotic prescription may play an important role in selecting MDR organisms, especially in the light of the high use of beta-lactam/beta-lactamase inhibitors and third generation cephalosporins in patients with COVID-19 [18].

In most of the reported cases of MDR *A. baumannii* infections in COVID-19 patients, high rates of mortality were observed [13, 45]. Our data confirm this trend; in fact, 75% of patients affected by a CR-Ab superinfections died, and CR-Ab was independently associated with 14-d mortality.

In our study, MRSA was detected in 6/64 (9.4%) infective episodes, mostly represented by BSIs. Our results are in line with those recently observed by Falcone et al., who found that MRSA accounted for 5.5% of superinfections episodes [14]. Conversely, these data are lower than the reported percentage of *S. aureus* isolates (including those resistant to methicillin) in patients with severe influenza, which accounted for up to 22–28% of total cases of pulmonary co- and superinfections and were associated with a high mortality rate [4, 46].

The low percentage of pulmonary Aspergillosis (1.9%) is in line with the data recently observed in our hospital context, where we found COVID-19 associated pulmonary aspergillosis (CAPA) in only a minority of ICU patients [47]. The incidence of CAPA is widely variable, ranging from less than 1% to more than 30% in critically ill patients [48, 49]; nevertheless, most data refer to the ICU setting. Accordingly, there is the need to elucidate the prevalence of this condition even in different settings, such as ours.

Our study presents some limitations: (i) this is a single-center retrospective observational study with a limited number of patients, that does not include all hospitalized patients; (ii) patients usually received empiric antibiotic therapy at admission, so that the prevalence of co-infections could be possibly underestimated, even if administering antibiotics was a common practice worldwide in that phase of pandemic and we reported similar prevalence of literature; (iii) lack of control group, such as COVID-19 patients hospitalized in ordinary wards or ICUs or patients without COVID-19; (iv) we were unable to investigate with specific microbiological analyses whether the isolation of CR-Ab was a cluster. Moreover, we developed this study in an emergency setting in the middle phase of the second wave, so we should acknowledge that the hospital surge during the pandemic may have affected patients’ outcome.

Nevertheless, our study offers important explanations on the prevalence and clinical impact of superinfections in COVID-19 patients hospitalized in a highly intensive care setting other than ICU. Accordingly, we believe that additional studies specifically focused on respiratory sub-intensive care units are warranted, in order to elucidate similitudes and differences, if any, between this special setting and the ICU.

## Conclusion

In conclusion, we showed that in a COVID-19 respiratory sub-intensive care unit, superinfections were common and represented an independent predictor of mortality. Conversely, co-infections had a low prevalence, supporting an antibiotic sparing strategy in the first phase of SARS-CoV-2 infection. Indeed, exposure to antibiotic therapy was independently associated with superinfections’ development. MDR organisms were found in almost half of patients with superinfections, and CR-Ab as the causative agent was associated with high mortality. For all these reasons, infection control rules and antibiotic stewardship programs represent critical points also in sub-intensive care units to limit the spread of MDR organisms.

## Supplementary Information

**Additional file 1**. **Supplementary Table 1**. Pathogens isolated for type and site of infection. **Supplementary Table 2**. Antimicrobial susceptibility testing of the 11 CR-Ab isolates causing superinfections.

## Data Availability

Data are available upon request from corresponding author.

## Abbreviations

ICU: Intensive Care Unit
MDR: Multidrug-resistant bacteria
ARDS: Acute respiratory distress syndrome
ABGs: Arterial blood gases
CPAP: Continuous positive airway pressure
CVC: Central venous access
NIMV: Non-invasive mechanical ventilation
XDR: Extensively drug-resistant pathogens
HFNC: High flow nasal cannula
MRSA: Methicillin-resistant *Staphylococcus aureus*
VRE: Vancomycin-resistant *Enterococcus faecium*
CR: Carbapenem resistant
TBA: Tracheobronchial aspirate
GM: Galactomannan
IQR: Interquartile ranges
CCI: Charlson Comorbidity Index
CAPA: COVID-19 associated pulmonary aspergillosis
