# Supplementary material for: Risk factors and effect on mortality of superinfections in a newly established COVID-19 respiratory sub-intensive care unit at University Hospital in Rome
Source: BMC Pulm Med. 2023 Jan 20;23:30. doi: 10.1186/s12890-023-02315-9 (PMC9854038; doi:10.1186/s12890-023-02315-9)
Supplement: Supplementary file 1 — Additional file 1. Supplementary Table 1. Pathogens isolated for type and site of infection. Supplementary Table 2. Antimicrobial susceptibility testing of the 11 CR-Ab isolates causing superinfections. [file 12890_2023_2315_MOESM1_ESM.docx]

|  |  |
| --- | --- |
| Co-infections at hospital admission | n (%) |
| *Mycoplasma pneumoniae* | 4 (100) |
| Superinfections | 64 (100) |
| Primary bloodstream infection | 23 (35.9) |
| Coagulase-negative *Staphylococci* | 5 (21.7) |
| Methicillin-resistant *Staphylococcus aureus* | 5 (21.7) |
| *Acinetobacter baumannii XDR* | 4 (17.3) |
| Methicillin-susceptible *Staphylococcus aureus* | 3 (13) |
| *Enterococcus faecalis* | 1 (4.3) |
| Vancomycin-resistant *Enteroccous faecium* | 1 (4.3) |
| *Enterobacter cloacae* | 1 (4.3) |
| *Candida albicans* | 1 (4.3) |
| *Candida parapsilosis* | 1 (4.3) |
| *Staphylococcus warneri* | 1 (4.3) |
| Hospital-acquired pneumonia | 19 (29.6) |
| *Acinetobacter baumannii XDR* | 6 (31.52) |
| *Pseudomonas aeruginosa* | 3 (15.7) |
| *Aspergillus fumigatus* | 3 (15.7) |
| *Klebsiella pneumoniae ESBL* | 1 (5.2) |
| *Klebsiella pneumoniae, no ESBL* | 1 (5.2) |
| *Klebsiella oxytoca* | 1 (5.2) |
| *Klebsiella pneumoniae, carbapenem resistant* | 1 (5.2) |
| *Aspergillus flavus* | 1 (5.2) |
| *Stenotrophomonas maltophilia* | 1 (5.2) |
| Not identified Gram negative bacillus | 1 (5.2) |
| Urinary tract infections | 18 (28.5) |
| *Escherichia coli* | 4 (22.2) |
| *Enterococcus faecalis* | 4 (22.2) |
| Vancomycin-resistant *Enteroccous faecium* | 3 (16.6) |
| *Klebsiella pneumoniae, ESBL* | 2 (11.1) |
| *Klebsiella pneumoniae, no ESBL* | 1 (5.8) |
| *Klebsiella pneumoniae, carbapenem resistant* | 1 (5.8) |
| *Acinetobacter baumannii XDR* | 1 (5.8) |
| *Pseudomonas aeruginosa* | 1 (5.8) |
| *Enterococcus faecium, not vancomycin resistant* | 1 (5.8) |
| Catheter-related bloodstream infections | 2 (3.1) |
| *Candida parapsilosis* | 1 (50) |
| *Staphylococcus epidermidis* | 1 (50) |
| Skin and soft tissues infections | 1 (1.5) |
| Methicillin-resistant *Staphylococcus aureus* | 1 (100) |
| *Clostridoides difficile* colitis | 1 (1.5) |

**Supplementary Table1.** Pathogens isolated for type and site of infection.

XDR: extended drug resistant; ESBL: extended spectrum beta lactamase; CR-BSI: catheter-related bloodstream infection.

**Supplementary Table2.** Antimicrobial susceptibility testing of the 11 CR-Ab isolates causing superinfections.

|  | **Amikacin**  **μg/mL** | **Gentamicin**  **μg/mL** | **Ampicillin/sulbactam**  **μg/mL** | **Meropenem**  **μg/mL** | **Colistin**  **μg/mL** | **Cefiderocol*** |
| --- | --- | --- | --- | --- | --- | --- |
| *Strain#1* | >32 | >8 | 16/8 | >16 | <2 | NP |
| *Strain#2* | >32 | >8 | >16/8 | >16 | <2 | S |
| *Strain#3* | >32 | >8 | 16/8 | >16 | <2 | S |
| *Strain#4* | ≥64 | ≥16 | NP | ≥16 | <0.5 | NP |
| *Strain#5* | >32 | >8 | 16/8 | >16 | <2 | S |
| *Strain#6* | >32 | >8 | >16/8 | >16 | <2 | S |
| *Strain#7* | ≥64 | ≥16 | NP | ≥16 | <0.5 | NP |
| *Strain#8* | ≥64 | ≥16 | NP | ≥16 | <0.5 | NP |
| *Strain#9* | ≥64 | ≥16 | NP | ≥16 | <0.5 | S |
| *Strain#10* | ≥64 | ≥16 | NP | ≥16 | <0.5 | NP |
| *Strain#11* | >32 | >8 | 8/4 | >16 | >4 | S |

*: cefiderocol susceptibility was performed by means of disk diffusion; results were given as S (susceptible) or R (resistant). NP: not performed.
